# Drowning in the Eastern Mediterranean region: a systematic literature review of the epidemiology, risk factors and strategies for prevention

**DOI:** 10.1186/s12889-022-13778-6

**Published:** 2022-08-03

**Authors:** Amy E. Peden, Ali Işın

**Affiliations:** 1 School of Population Health, UNSW Sydney, Samuels Building, UNSW Sydney, Kensington, NSW 2052 Australia; 2 College of Public Health, Medical and Veterinary Sciences, James Cook University, Townsville, Queensland 4811 Australia; 3 Department of Coaching Education, Faculty of Sports Sciences, Akdeniz University, 07070 Antalya, Turkey; 4 Institute of Health Sciences, Akdeniz University, 07070 Antalya, Turkey

**Keywords:** Water safety, Data, Policy, Education, Mortality, Morbidity

## Abstract

**Introduction:**

Globally, drowning is a significant cause of preventable mortality and morbidity. The Eastern Mediterranean region (EMR) comprises 22 countries of extreme disparity in income and is a region impacted by conflict and migration. We systematically review literature published on drowning in the EMR.

**Methods:**

Peer-reviewed literature (limited to original research) was identified using Embase, PubMed, Scopus, SportsDiscus, and Web of Science databases. Literature was independently dual screened at title/abstract and full text stages with dual data extraction (20% of included studies). Studies were included if they reported epidemiology, risk/protective factors and/or prevention strategies for drowning (unintentional and intentional; fatal and non-fatal) of residents, tourists or migrants in the EMR. Literature was assessed against the [Australian] National Health and Medical Research Council’s Levels of Evidence.

**Results:**

Seventy-two studies were included in this review (epidemiology 68 studies; risk/protective factor 13 studies; prevention strategies 19 studies). Iran (*n* = 27), Saudia Arabia (*n* = 11) and Pakistan (*n* = 10) recorded the largest number of dedicated studies. Studies predominately focused on unintentional drowning. Ninety-two percent of included studies (*n* = 66) were ranked as being low evidence (level IV). The majority of studies explored drowning among children and adolescents (0–19 years). All-age fatal drowning rates varied from a low of 0.48 per 100,000 (United Arab Emirates; 2002; Ministry of Health death registry data) to a high of 18.5 per 100,000 (Egypt; 2014–15; WHO mortality database). Commonly identified risk factors included being male, young age, submersion time and resident status. Common prevention strategies public education, lifeguard supervision, and cardiopulmonary resuscitation.

**Discussion:**

Gaps in understanding of drowning burden in some countries within the region, as well as region-wide risk factor identification for adult drowning, intentional and migration-related drowning, impair the ability of nations to advance drowning prevention. There is a need for investment in implementation and evaluation of drowning prevention interventions in the EMR.

**Conclusion:**

Drowning is a significant cause of mortality and morbidity in the EMR. The recent UN declaration on global drowning prevention may provide the impetus to invest in drowning prevention research, policy, and advocacy with the aim of reducing drowning-related harms in the EMR.

**Trial registration:**

Registration number: #CRD42021271215.

**Supplementary Information:**

The online version contains supplementary material available at 10.1186/s12889-022-13778-6.

## Introduction

Globally, drowning is a significant cause of preventable harm [1] which has recently been acknowledged in a United Nations (UN) General Assembly resolution [2]. In 2017, the Global Burden of Disease (GBD) Study estimated 295,000 lives lost to drowning [3]. More recently, the World Health Organization (WHO) estimated 236,000 lives lost globally in 2019 [4]. However, both estimates are likely to be significant underreports due to the exclusion of fatal unintentional drowning due to transportation and disaster-related causes. The exclusion of such cases is shown to underreport drowning by between 40 and 60% in some high-income countries (HICs) [5–7], with the impact likely to be significantly higher in low- and middle-income countries (LMICs). In addition, there is limited global data on the impact of intentional drowning as a cause of death [8, 9].

Our understanding of drowning is further limited due to a lack of data globally on non-fatal drowning. Drowning is defined as the process of experiencing respiratory impairment due to submersion or immersion in liquid, with outcomes being death, morbidity or no morbidity [10]. However, a lack of uniform classifications for non-fatal drowning and a lack of data has hampered global estimates [11].

The Eastern Mediterranean Region (EMR) of the WHO comprises 21 member states and the occupied Palestinian territory (including East Jerusalem), with a population of nearly 679 million people [12]. The EMR spans countries from North Africa to Western Asia and the unique geographical, social and economic characteristics of each country yield differing burden of injuries and violence [13]. It is a region that has significant disparities in income levels and has been heavily impacted by conflict [14] and migration, with untold loss of life at sea [15].

Drowning has been identified as an issue in the EMR. A previous review exploring injuries and violence in the EMR reported that unintentional drowning accounted for 6% of all injury deaths in 2012, slightly lower than the global burden at 7% [13]. Unintentional drowning appeared in the top 10 causes of years of life lost in 2013 for EMR countries of Jordan (ranked 7th), Oman (ranked 9th) and Qatar (ranked 10th) [16]. Suicidal drowning has also been identified as an issue of concern in the EMR, accounting for 3.1% of all suicide deaths in the region, with a slightly higher proportion among females (5.0%) than males (4.5%) [17].

Despite previously published location-based literature reviews exploring drowning in low and middle income countries [18], the African continent [19] and individual countries such as India [20] and Singapore [21], no previous systematic literature review has explored drowning in the EMR. As such, the aim of this literature review is to identify the published literature exploring fatal and non-fatal drowning (regardless of intent) in the EMR, with a focus on the epidemiology reported, risk factors identified, and prevention strategies recommended.

## Methods

The protocol for this systematic review was prospectively registered with PROSPERO (#CRD42021271215) and followed the Preferred Reporting Items for Systematic Reviews and Meta-Analysis (PRISMA) guidelines to identify, screen, determine eligibility and include studies [22].

Peer-reviewed literature, limited to original research, was identified via searches of the Embase, PubMed, Scopus, SportsDiscus and Web of Science databases. In addition, we also searched the Google Scholar website to identify additional articles not found through database searches. Google Scholar results were searched until 10 pages of nil results. Database searches were run on 11 August 2021, with the Google Scholar search run on 19 September 2021. The full search strategies used for each database and Google Scholar can be found in Table S1. Additionally, reference lists of relevant excluded studies were searched for additional literature that may not have been identified prior. As a result of this process four additional studies were identified.

Studies were included if they explored drowning in the EMR as defined by the World Health Organization (WHO) [12]. The EMR is comprised of the following member states and territories: Afghanistan, Bahrain, Djibouti, Egypt, (Islamic Republic of) Iran, Iraq, Jordan, Kuwait, Lebanon, Libya, Morocco, Oman, Pakistan, Palestine (Occupied Palestinian Territory), Qatar, Saudi Arabia, Somalia, Sudan, Syrian Arab Republic, Tunisia, United Arab Emirates, and Yemen. Studies exploring drowning were included if they reported fatal or non-fatal drowning of any intent (i.e., unintentional, intentional, undetermined intent). We included in this broad definition drowning that was recreational in nature, occupational-related drowning, conflict-related drowning and migration-related drowning. We included studies reporting drowning of non-residents of the EMR if drowning occurred in EMR. We excluded non-human studies, studies focused on the forensic diagnosis of drowning, or the pathophysiology of drowning. We also excluded studies exploring drowning among EMR-born people who reside (and drowned) in non-EMR countries (e.g., EMR residents who emigrated to Scandinavia). Literature published from inception to 11 August 2021 was included in the study.

Literature was screened using Covidence literature screening software [23]. Independent dual screening was performed at title and abstract stage, and again at full text stage, with conflicts resolved via consensus between the two authors. Data extraction was performed using a custom-built Microsoft Excel spreadsheet. Independent data extraction was conducted by both authors, with dual extraction of 20% of included studies.

Studies reporting the epidemiology of drowning were extracted as numbers, proportions or rates per 100,000 for each population reported (i.e., overall, by sex, by age group, by year etc). Where drowning rates were not presented but could be derived (i.e., drowning cases and population data presented), these were manually calculated. Drowning was characterised by outcome (fatal, non-fatal, both, not specified), and intent (unintentional, intentional, both, not specified) and explored at a total population level, as well as by age group and sex. Trends in all-age drowning rates were calculated using the linear trend function in Excel.

Risk/protective factors were defined if statistical tests identified a significant link between the factor and risk of drowning or drowning outcome (i.e., chi square tests of significance, odds ratio, relative risk). The free text description of risk/protective factors was coded by both authors via consensus (see Table S2 for method). Free text of prevention strategies was coded by both authors via consensus (see Table S3 for method). Prevention strategies were extracted if proposed, implemented and/or evaluated. Prevention strategies were coded as primary, secondary or tertiary prevention [24] and against the corresponding level within the Hierarchy of Control [25]. We also recorded if the prevention strategy involved multi-sectoral action (as recommended by the WHO [26]) and if so, which sectors were involved (i.e., health, transport, maritime safety, tourism, disaster preparedness etc). We also assessed whether the prevention strategy mentioned in the literature, aligned to the six selected interventions and four cross-cutting implementation strategies for the prevention of drowning, as recommended by the WHO [26].

Included studies were assessed against the [Australian] National Health and Medical Research Council’s Levels of Evidence [27]. Levels of evidence range from Level I (a systematic review of Level II studies [randomised controlled trial]) to Level IV (case studies with either post-test or pre-test/post-test outcomes) [27]. Income levels of countries represented in included studies were assessed using the World Bank open data country profiles [28].

## Results

**Fig. 1:**
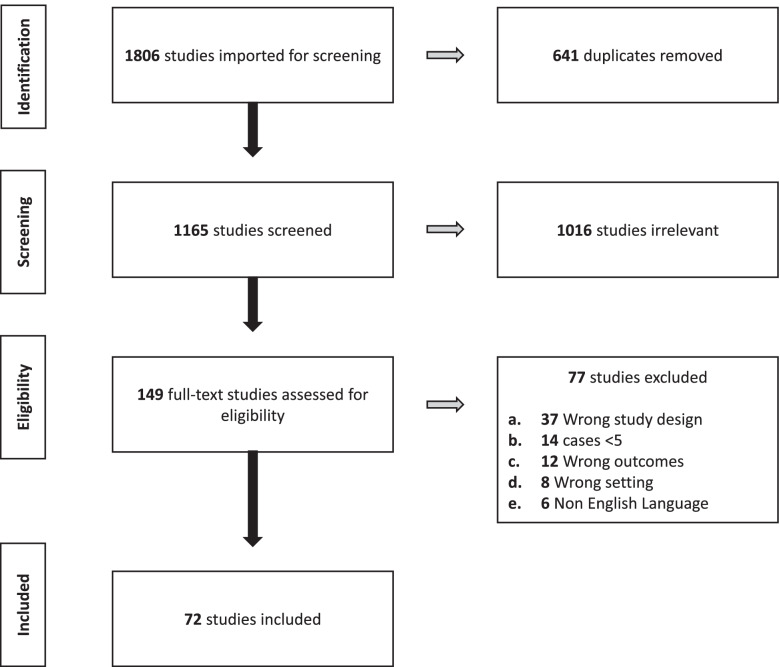
PRISMA flow chart

A total of 1806 studies were identified via database searches and Google Scholar. After the removal of duplicates (*n* = 641), 1165 studies were screened for inclusion by title and abstract. Of these, 1016 studies were deemed irrelevant (87.2%). A total of 149 full text studies were screened for eligibility. After the removal of 77 (51.7%) studies at full text review, a total of 72 studies were included for data extraction (Fig. 1).

### Study characteristics

Included studies were published between 1975 [29] and 2021 [30–32]. Studies most commonly reported data on drowning from Iran (27 studies [33–59]), Saudia Arabia (11 studies [31, 32, 60–68]) and Pakistan (10 studies [69–78]). There were 3 studies which reported on multiple countries within the EMR [79–81] and 3 studies which reported on the EMR as a whole [3, 82, 83] (Table 1).

**Table 1:** Included studies by country and exploration of drowning by epidemiology, risk factors and prevention strategies

Region/Country	World Bank Income Level	Total number of studies	% of total studies
Eastern Mediterranean Region	–	3 studies [3, 82, 83]	4.2%
Afghanistan	Low income	1 study [79]	1.4%
Bahrain	High income	3 studies [30, 84, 85]	4.2%
Djibouti	Lower middle income	–	–
Egypt	Lower middle income	4 studies [80, 81, 86, 87]	5.6%
Iran	Lower middle income	27 studies [33–59]	37.5%
Iraq	Upper middle income	3 studies [79, 88, 89]	4.2%
Jordan	Upper middle income	3 studies [90–92]	4.2%
Kuwait	High income	–	–
Lebanon	Upper middle income	1 study [81]	1.4%
Libya	Upper middle income	–	–
Morocco	Lower middle income	1 study [80]	1.4%
Oman	High income	–	–
Palestine	Lower middle income^a^	2 studies [81, 93]	2.8%
Pakistan	Lower middle income	10 studies [69–78]	13.9%
Qatar	High income	3 studies [94–96]	4.2%
Saudia Arabia	High income	11 studies [31, 32, 60–68]	15.3%
Somalia	Low income	–	–
Sudan	Low income	1 study [29]	1.4%
Syria	Low income	–	–
Tunisia	Lower middle income	2 studies [81, 97]	2.8%
United Arab Emirates	High income	2 studies [98, 99]	2.8%
Yemen	Low income	–	–

A study may cover more than one country. All percentages are as a proportion of the final 72 included articles. ^a^ Classified as West Bank and Gaza in World Bank data

Twenty-seven studies (37.5% of all included studies) reported data on a national level, with the remaining studies exploring data at a sub-national level. Thirty-six studies (50.0%) reported data from both urban and rural areas, 18 studies (25.0%) reported data from urban areas only and 18 studies (25.0%) examined drowning in rural areas only. When exploring outcome, 42 studies (58.3%) explored fatal drowning, 10 studies (13.9%) explored non-fatal drowning and 20 studies (27.8%) explored both fatal and non-fatal drowning. Twenty-eight (38.9%) studies reported unintentional drowning, 3 studies (4.2%) reported intentional drowning and 9 studies (12.5%) reported drowning regardless of intent. The remaining 32 studies (44.4%) did not specify the intent of the drowning cases reported. The majority of included studies were ranked as being of low evidence (level IV; 66 studies; 91.7%). The characteristics of all included studies can be viewed in full in Table S4.

### Epidemiology

**Fig. 2:**
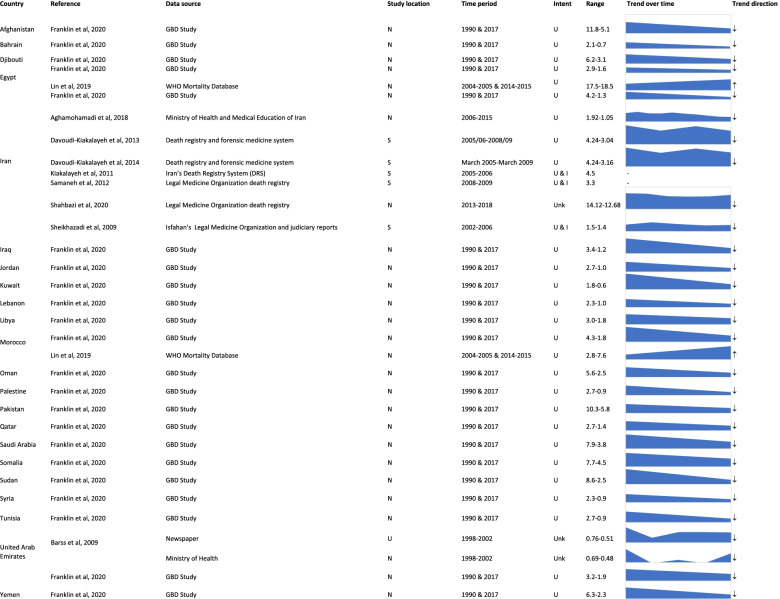
Rate and trends over time of all-age fatal drowning by country, Eastern Mediterranean Region. *GBD* Global Burden of Disease, *N* National, *S* Sub-national, *U* Unknown. *U* Unintentional, *I* Intentional, *Unk* Unknown

Sixty-eight (94.4%) included studies reported the epidemiology of drowning in the EMR. There were 10 studies [3, 34, 40, 43, 50, 54–56, 80, 98] which reported all-age fatal drowning rates. Data sources ranged from GBD study [3] and WHO mortality database [80], to death registry and newspaper reports [98] and the majority of studies reported unintentional drowning (Fig. 2).

All-age fatal drowning rates ranged from a low of 0.48 per 100,000 in the United Arab Emirates in 2002 as per Ministry of Health death registry data [98], to a high of 18.5 per 100,000 population in Egypt in 2014–15 [80]. Drowning rates in all countries reported a downward trend, with the exception of Morocco and Egypt, which WHO mortality data indicated an increasing trend in fatal unintentional drowning between 2004 and 05 and 2014–15 [80] (Fig. 2).

There were a further 11 studies [35, 40, 43, 44, 50, 52, 54–56, 83, 93] which reported fatal drowning rates by sex and/or age group, the majority of these studies came from Iran. When exploring the EMR as a whole, data from 2004 for children and adolescents aged 0–19 years indicated the highest fatal drowning rate among 1–4 year-olds (9.0 / 100,000 population). For females, the rate was highest among those aged < 1 year of age (7.0 per 100,000) and for males, in the 1–4 years age group (11.0 per 100,000) [83]. Outside of the child and adolescent (0–19 years) age group, the highest drowning rates were reported in Iran among 20–24 year-olds between 2002 and 2007 (17.0 per 100,000 population) [35] (Table 2).

**Table 2:** Rates of fatal drowning by age group and sex, Eastern Mediterranean Region

Region/ Country	Study year(s) (reference(s))	0–4		5–9	10–14	15–19	20–24	25–29	30–34	35–39	40–44	45–49	50–54	55–59	60–64	65–69	70+
Both males and females																	
EMR	2004 [83]	7.7 (< 1 yr)	9.0 (1-4 yr)	5.9	5.2	7.3											
Iran	2002–2007 [35]	1.5		3.1	9.4	16.1	17.0	9.4	6.1	4.3	4.1	2.8	2.6	1.0	1.4	2.4	1.2
	2005–06 [40, 43]	2.75			6.12		4.08									3.90	
	2006–07 [40, 43]	2.61			5.40		2.40									3.07	
	2007–08 [40, 43]	2.47			5.40		4.00									2.82	
	2008–09 [40, 43]	2.08			2.10		3.80									2.80	
	2006–2006 [50]	1.0 (0–15 years)					3.55 (16+ years)										
	2005 [52]	4.3 (< 1 yr)	4.2 (1–4 yrs)	1.8	1.9												
	2002–2006 [56]	1.8		1.8		3.0		1.1		0.5		0.5		0.8		0.8	
Palestine	2001–2003 [93]	2.7		0.8	0.7	1.6											
Females																	
EMR	2004 [83]	7.0 (< 1 yr)	6.8 (1–4 yrs)	4.3	2.9	3.3											
Iran	1990–2015 [44]	5.2															
	2002–2006 [56]	1.4		0.6		0.4		0.3		0.1	0.2			0.2		0.3	
	2005–2006 [50]	0.68															
	2008–2009 [54]	0.54															
	2008–2009 [54]	–		0.5		1.2		0.3			–			–			–
	2013 [55]	4.83															
	2014 [55]	4.43															
	2015 [55]	2.98															
	2016 [55]	4.31															
	2017 [55]	2.92															
	2018 [55]	4.21															
Males																	
EMR	2004 [83]	8.4 (< 1 yr)	11.2 (1–4 yrs)	7.5	7.4	11.0											
Iran	1990–2015 [44]	7.5															
	2002–2006 [56]	2.1		2.9	5.6		1.8			0.8		0.9		1.3		1.2	
	2005–2006 [50]	3.86															
	2008–2009 [54]	6.2															
	2008–2009 [54]	3.1		3.9		11.1		4.7			2.5			1.5			–
	2013 [55]	23.22															
	2014 [55]	22.97															
	2015 [55]	19.85															
	2016 [55]	21.47															
	2017 [55]	19.70															
	2018 [55]	20.91															

Data on non-fatal drowning were limited. Several studies identified the significant non-fatal drowning burden among young children less than five [32, 51, 67]. By contrast, a study from Saudi Arabia reported almost half (43.1%) of all non-fatal drowning cases in children 1–14, occurring among school age children (7–14 years of age) [64]. While males typically report significantly higher fatal drowning rates, among the non-fatal cases reported, the sex split was proportionately more similar. A study of non-fatal drowning among children 0–14 years of age in Saudi Arabia identified 40% of cases were of girls [32]. Among the all-age population, a study from northern Iran highlighted females accounted for 20.2% of all non-fatal drownings from 2007 to 2008 [42]. A study from Pakistan reported similar findings, with females accounting for 27.9% of all emergency department (ED) presentations for non-fatal drowning [69]. A study of hospital admissions in Iran between 1995 and 2005, found females accounted for 33% of non-fatal drowning-related admissions [51].

Two studies also reported fatal drowning rates by rurality. An all-age study of drowning in Iran between 2005 and 2006, found a higher rate of fatal drowning in rural areas (2.6 per 100,000 population) than in urban areas (1.8) [50]. A study of drowning in Iran among children 0–14 years in 2005 found even higher differences in fatal drowning rates by rurality (rural residence: 4.7; urban residence: 1.3) [52]. One study also reported rates of fatal drowning by aquatic location. In northern Iran, fatal drowning in coastal locations occurred at a rate of 2.48 per 100,000 population, compared with 1.5 for rivers, 0.15 for canals and 0.35 for other natural water locations [50].

### Risk/protective factors

There were 13 studies which reported on 18 unique risk/protective factors associated with drowning in the EMR. The majority of studies were conducted among children and adolescents, with just 4 studies examining the all-age population, all from Iran [40, 42, 55, 59]. One study identified factors associated with the self-reported behaviours of parents of infants in Saudi Arabia [68] (Table 3).

**Table 3:** Coded risk and protective factors for fatal and non-fatal drowning in the Eastern Mediterranean Region

Risk/Protective Factor	Specific detail	Drowning outcome	Study population	Country (Reference)
Absence of vital signs	Absence of vital signs	Fatal	0–15 years	Iran [51]
	Absence of vital signs in emergency room	Brain death or severe neurological disease	1–13 years	Saudi Arabia [63]
Accidental intent	More likely to be of accidental than due to suicide or homicide	Fatal	0–18 years	Iran [58]
Adult supervision	Unsupervised by adults	Fatal	0–13 years	Saudi Arabia [60]
Age	1–4 years age group	Fatal and non-fatal	0–13 years	Bahrain [30]
	Children ≤5 years	Fatal	0–17 years	Saudi Arabia [31]
	Children 1–4 years	Fatal	0–18 years	Iran [58]
Blood sugar level	Blood sugar of ≥10 mmol/L	Brain death or severe neurological disease	1–13 years	Saudi Arabia [63]
Death at scene	Location of death more likely to be at the scene for drowning	Fatal	0–18 years	Iran [58]
Delay in initiating resuscitation	Delay in initiating resuscitation	Fatal or leading to neurological damage	0–14 years	Saudi Arabia [32]
Glasgow Coma Scale (GCS) score	High GCS score upon admission ^a^	Complete recovery	0–14 years	Saudi Arabia [32]
	High GCS score upon end of care ^a^	Complete recovery	0–14 years	Saudi Arabia [32]
	GCS score < 5	Fatal	0–15 years	Iran [51]
	GCS score ≤ 4	Brain death or severe neurological disease	1–13 years	Saudi Arabia [63]
Length of hospital stay	Decreased length of stay ^a^	Complete recovery	0–14 years	Saudi Arabia [32]
Mother with helper or older children	Self-reporting leaving infant unattended in the bathtub	Non-fatal	Parents of infants	Saudi Arabia [68]
Non-lifeguarded areas	Non-lifeguarded areas	Fatal	All ages	Iran [59]
pH level	pH < 7.2	Fatal	0–15 years	Iran [51]
	Arterial pH of ≤7	Brain death or severe neurological disease	1–13 years	Saudi Arabia [63]
Resident status	Tourists more likely to drown than local residents	Revived (subsequently fatal)	All age	Iran [59]
	Tourists drowning in the sea	Fatal	All age	Iran [59]
	Local residents	Fatal	All age	Iran [59]
Seasonality	Winter and Autumn months	Fatal	0–13 years	Saudi Arabia [60]
Sex	Males	Fatal	All age	Iran [42]
	Males	Fatal	All age	Iran [40]
	Males	Fatal	All age	Iran [55]
	Males	Fatal	0–15 years	Iran [51]
	Males	Fatal	0–18 years	Iran [58]
	Males	Fatal	1–5 years	Qatar [94]
	Males	Fatal	0–17 years	Saudi Arabia [31]
	Females aged ≤14 years	Fatal	All age	Iran [59]
Submersion time	Longer submersion time	Fatal	0–13 years	Saudi Arabia [60]
	Submersion time > 5 minutes	Fatal	1–13 years	Saudi Arabia [63]
	Shorter submersion time (mean 3.5 minutes) ^a^	Fatal or leading to neurological damage	0–14 years	Saudi Arabia [32]
Body temperature	Temperature ≥ 36 degrees ^a^	Recovery	0–15 years	Iran [51]
Washing containers	Outcome worse when compared to pools	Fatal	0–13 years	Saudi Arabia [60]

^a^denotes protective factor

The majority (15 factors including low Glasgow Coma Scale [GCS] score) were identified as factors which increased risk of both fatal or non-fatal drowning with poor outcomes (such as severe neurological disease or brain death). Commonly identified risk factors included being male [31, 40, 42, 51, 55, 58, 94], young age [30, 31, 58], submersion time [60, 63] and resident status (both tourists and local residents) [59]. There were several factors identified in the literature as being protective for drowning outcome. These were a high GCS score upon admission and upon end of care [32], decreased length of hospital stay [32], shorter length of time submersed [32] and a body temperature of ≥36 degrees [51] (Table 3).

### Prevention of drowning

A total of 19 studies reported 17 unique strategies for the prevention of drowning in the EMR (Table 4). Common strategies detailed in included studies were public education (mentioned in 7 studies across 4 countries), lifeguard supervision (mentioned in 6 studies across 3 countries), and cardiopulmonary resuscitation (CPR) and first aid (mentioned in 6 studies across 4 countries).

**Table 4:** Drowning prevention strategies documented in included literature

Prevention strategy	Country	Primary, secondary or tertiary prevention	Proposed (P), implemented (I) or evaluated (E)	Hierarchy of Control	Strategy span multiple sectors? (Y/N)	If yes, which sectors?	Align to WHO recommended interventions and Strategies? (Y/N)
Beach safety assessment	Jordan [90]	Secondary	P	Administrative	N	–	N
Child Supervision	Bahrain [30] Saudi Arabia [32]	Primary	P	Administrative	N	–	Y- Provide safe places away from water for pre-school children, with capable child care
	Tunisia [97]	Primary	P	Administrative	Y	Education	
CPR and First Aid	Bahrain [85]	Tertiary	P	Administrative	Y	Maritime	Y - Train bystanders in safe rescue and resuscitation
	Iran [40]	Tertiary	E ^b^	Administrative	Y	Health	
	Jordan [90]	Tertiary	I	Administrative	Y	Health	
	Saudi Arabia [60, 32]	Tertiary	P	Administrative	Y	Health	
	Saudi Arabia [66]	Tertiary	E #	Administrative	Y	Health	
Developing a national water safety strategy	Bahrain [84]	Primary	P	Administrative	N	–	Y – Develop a national water safety plan
Lifeguard supervision	Bahrain [85]	Primary	P	Administrative	Y	Tourism	N
	Iran [42, 43, 49] Pakistan [71]	Primary	P	Administrative	N	–	
	Iran [40]	Primary	E ^a^	Administrative	N	–	
Lifeguard supervision (female lifeguards)	Pakistan [75]	Primary	P	Administrative	N	–	
Lifeguard supervision (multilingual lifeguards)	Egypt, Lebanon, Palestine and Tunisia [81]	Primary	P	Administrative	Y	Tourism	
Lifeguard supervision (police presence at beaches)	Pakistan [75]	Primary	P	Administrative	Y	Police	
Personal flotation devices	Bahrain [30]	Primary	P	Administrative	N	–	N
Public education	Iran [42, 43] Palestine [93] Saudi Arabia [60]	Primary	P	Administrative	Y	Media	Y – Strengthen public awareness of drowning through strategic communications
	Pakistan [76] Palestine [93]	Primary	P	Administrative	Y	Health	
	Iran [40]	Primary	E*	Administrative	Y	Health & Media	
Restrict access to water	Bahrain [30] Pakistan [73]	Primary	P	Isolation	N	–	Y – Install barriers controlling access to water
	Iran [43]	Primary	P	Isolation	Y	Local government	
	Iran [40]	Primary	E ^a^	Elimination	Y	Provincial government	
Spa regulations	Egypt, Lebanon, Palestine & Tunisia [81]	Primary	P	Administrative	Y	Tourism	N
Signage	Jordan [90]	Primary	I	Engineering	N	–	N
Swimming and Water Safety Training	Bahrain [30] Iran [42, 43, 49]	Primary	P	Administrative	N	–	Y – Teach school-age children swimming and water safety skills
Vehicle modification	Afghanistan and Iraq [79]	Primary	I	Engineering	Y	Ministry of Defence	N
Vehicle operational procedures	Afghanistan and Iraq [79]	Primary	I	Administrative	Y	Ministry of Defence	N
Vehicle-related water safety training	Afghanistan and Iraq [79]	Secondary	I	Administrative	Y	Ministry of Defence	N

*P* Proposed, *I* Implemented, *E* Evaluated, *WHO* World Health Organization. ^a^(Fatal drowning rate 4.24 / 100,000 residents at baseline to 3.04 at end line) ^b^(Parental knowledge of first aid)

Of the 17 unique prevention strategies identified in the literature, the vast majority were proposed, with just five strategies (CPR and First Aid and signage in Jordan [90] and vehicle modification, vehicle operational procedures and vehicle-related water safety training for US troops in Afghanistan and Iraq [79] (Table 4).

Two studies reported the evaluation of drowning prevention interventions. One study in Saudi Arabia assessed parental knowledge of first aid. This study indicated 64.3% knew to lay the victim in a left lateral position. In a nonresponsive not breathing drowning victim, 90.5% knew to start chest compressions and mouth to mouth ventilation. Sixty-six percent (65.6%) knew not to slap a victim on the back [66]. Another study explored the impact on fatal drowning rates of a package of interventions in two coastal areas of Iran, including CPR and first aid training, public education, elimination of water reservoirs and increased lifeguard supervision. This study found the fatal drowning rate fell from 4.24 / 100,000 residents at baseline to 3.04 at end line [40] (Table 4).

The majority of the prevention strategies in the included literature were low on the hierarchy of control, with all but three (signage and vehicle modification [engineering] and restricting access to water [elimination or isolation]) being administrative in nature. Several strategies were identified as being multi-sectoral in nature involving sectors such as education, maritime, health, police, defence, media and tourism, as well as land managers such as local and provincial government (Table 4).

Six of the strategies proposed align with the six selected interventions and four cross-cutting implementation strategies outlined by the WHO. These include the provision of safe places away from water for pre-school children with capable childcare, train bystanders in safe rescue and resuscitation, develop a national water safety plan, strengthen public awareness of drowning through strategic communications, install barriers controlling access to water and teach school-aged children swimming and water safety skills (Table 4).

## Discussion

Drowning is a preventable cause of mortality and morbidity in all regions of the world [3], including in the EMR. While significant research has been undertaken to date in countries such as Iran, Saudia Arabia and Pakistan, there remains a dearth of literature on drowning in Yemen, Syria, Somalia, Oman, Libya, Kuwait, and Djibouti, aside from modelled data available via the GBD Study. This review has also identified the need for further research quantifying drowning among adults, intentional drowning burden and migration-related drowning. Risk factor identification was predominately limited to children and there is extremely limited data on the implementation and evaluation of prevention strategies.

The EMR is a diverse region comprising a mix of high and low-and middle-income countries [28]. As such, there was wide variation in the rates of all-age fatal drowning across the region reported in included studies. Rates varied from a low of 0.48 per 100,000 population for unintentional drowning via national Ministry of Health data in the United Arab Emirates in 2002 [98], to a high of 18.5 per 100,000 population for national unintentional drowning in Egypt in 2014–15 [80], using the WHO Mortality Database. Similarly, systematic reviews of drowning in the African region [19] and in low and middle income countries [18] have also reported large variation in rates of fatal drowning. Inconsistencies in data collection makes data synthesis extremely challenging. In addition, there were several countries in the EMR where no studies had been conducted and no data on drowning were available in the peer reviewed literature beyond GBD study modelled data. We echo the call of others, on the need for further research on the issue of drowning in low- and middle-income countries [100, 101].

The existence and availability of accurate and timely data on drowning in many countries in the region is likely impacting country-level analyses of drowning [65]. Several studies included in this review highlighted data challenges such as differences in reporting between data sources in Iran [50] and underreporting of drowning in Ministry of Health death data in the United Arab Emirates, when compared to newspaper reports [98]. Such findings underscore the importance of strengthening existing surveillance systems or developing new systems for consistent and detailed capture of drowning cases [19]. There is a need for prioritisation and investment in, country level drowning registries [102] to aid in the quantification of burden, identification of at risk groups and development, implementation and evaluation of drowning prevention interventions, within the framework of a National Water Safety Plan as recommended by the WHO [26]. This should include all cases of fatal and non-fatal drowning, regardless of intent.

The drowning literature for the EMR published to date identified in this review, has largely explored unintentional fatal drowning. The two key data sources included in this review, the GBD Study [3] and the WHO Mortality Database [80], define unintentional drowning using ICD codes W65–74 (accidental drowning and submersion). This narrow definition of drowning does not capture unintentional drowning due to water transport and disaster events, which has been shown to underreport drowning by between 40 and 60% in selected high-income countries [5–7]. Therefore, the declining rates of all-age drowning fatalities reported in this review must be interpreted with caution. Broader inclusion of drowning cases is another benefit of establishing national or regional drowning registries. Included studies also overwhelmingly focused on drowning among children and adolescents, with a need to identify drowning burden, risk factors and prevention strategies for adults and older adults, given an ageing population globally [103, 104].

There were no studies exploring migration-related drowning and only one study exploring conflict-related drowning [79] despite the EMR being a region that is significantly impacted by both issues [105]. Migration in particular is likely to be a significant contributor to the drowning burden in the EMR, with the Missing Migrant project indicating drowning is a leading cause of death during travel along migratory routes [15]. In addition, several studies conducted in countries outside the EMR were identified in initial searches, which pointed to an increased drowning risk among migrants from EMR countries [106, 107]. Although outside the scope of this review, such studies highlight a lack of drowning prevention interventions within the EMR, increasing drowning risk for both residents of the EMR and migrants originating from the EMR.

Being male was a commonly identified risk factor for drowning in the EMR, as has been found elsewhere [108–110]. The exception being an all-age study from Iran, which found females aged ≤14 years to be at increased risk of fatal drowning [59]. There may be cultural or other reasons behind this anomalous finding that should be further explored. Little research on the impact of seasonality has been conducted in the EMR, although one study from Saudi Arabia, identified the Winter and Autumn months as being a risk factor for drowning [60]. This differs from many other studies reporting increased drowning risk with periods of warmer weather [111–113]. This may be due to extreme summer temperatures in Saudi Arabia impacting patterns of exposure with water. With a changing climate forecast to have significant impacts on drowning risk [114], the differing profile of drowning risk for countries already prone to extreme temperatures in the EMR will likely need to be considered when developing preventive approaches in the near future.

Overwhelmingly however, risk factors for drowning in the EMR were identified based on studies of drowning in children and adolescents. While young people account for a significant proportion of the global burden of drowning [1, 3], there is a need to better understand risk factors for adult and older adult drowning, as well as deeper exploration of location and activity-based risk factors to inform prevention efforts. Such challenges in the identification of risk factors have been previously highlighted in systematic reviews of drowning in the African region [19] and in low and middle income countries [18]. The authors of these studies, as do we, call for detailed data to enable risk factor identification to target preventive approaches.

Evidence surrounding prevention interventions identified in the literature was extremely poor, with extremely limited implementation and evaluation of proposed interventions. Of note however, a study exploring the effect of a package of interventions in coastal regions of Iran, indicated positive reductions in fatal drowning rates [40]. The study does, however, highlight the complexity of implementation with a diversity of sectors and organisations involved, as well as the need for accurate and timely data to evaluate impact [40]. Other literature reviews of drowning have also highlighted the dearth of studies exploring the prevention of drowning, compared to the larger bodies of literature on drowning epidemiology and risk factors [18, 19, 104, 115]. Although conducting studies to identify the effectiveness of drowning prevention interventions are challenging, it represents one of the greatest needs for the drowning prevention sector globally [26]. In the absence of data to identify effectiveness of prevention interventions, validation of expert opinion on drowning prevention interventions via a Delphi process, may be an option, as has been used in other areas of drowning where evidence is lacking [116, 117].

This study is the first of its kind to systematically report on literature exploring the epidemiology, risk factors and prevention strategies for drowning in the EMR. However, the finding of this systematic literature review should be considered within the context of some limitations. This review was conducted in the English language only and may have therefore excluded studies published in languages other than English. This review included primary studies published in peer-reviewed literature only. There may also be relevant information on the issue of drowning and its prevention within the EMR published in the grey literature. The identification of studies is based on the search strategy used. A different search strategy would yield differing results. The resulting included literature is based on the two authors application of the inclusion and exclusion criteria, though dual screening and partial dual extraction methods were utilised to reduce any bias or human error. The World Bank income level attributed to the country is from data from 2019, a country’s income level may have been changed since the included study was conducted. There is a paucity of data on drowning in the EMR outside of Iran, Saudi Arabia, and Pakistan (66.7% of all included articles). It is not known if the risk factors and prevention strategies identified in included studies can be extrapolated to the broader EMR.

## Conclusion

Drowning is a significant cause of mortality and morbidity in the EMR; a diverse region that faces significant conflict and migration-related public health challenges. Although many included studies report declining drowning rates, there is a need to examine the full burden of drowning to determine if reductions in unintentional drowning (i.e., ICD codes W65–74) also hold true for water transport and disaster-related drowning. Despite reported reductions, drowning rates remain significant in countries such as Egypt, Afghanistan, and Iran, and among young children and adolescents. However, there is a dearth of research on drowning in the region outside of Iran, Saudia Arabia and Pakistan and on drowning in adults. Additionally, intentional drowning, non-fatal drowning, and the drowning burden associated with migration are poorly researched within the region. Data gaps impair understanding of risk factors and the ability of nations to develop water safety plans to reduce drowning, as recommended by the WHO. Many included studies rely on a single source of data (GBD Study) which exclude water transport and disaster-related drowning. Investment in the establishment of national (or regional) drowning registries will enhance accurate surveillance and monitoring as well as facilitate research to better understand causal factors and impact of preventive approaches. The recent UN Declaration on Global Drowning Prevention provides an opportunity to invest in drowning prevention research, policy, and advocacy with the aim of reducing the drowning-related burden in the EMR. Such efforts must be prioritized to end this preventable loss of life in the region.

## Supplementary Information

**Additional file 1: Table S1.** Search Strategy. **Table S2.** Description of risk/protective factors, their measure of significance and coding methodology. **Table S3.** Prevention strategies as described in included studies and coding methodology used, Eastern Mediterranean Region. **Table S4.** Characteristics of included studies.

## Data Availability

All data generated or analysed during this study are included in this published article (and its Supplementary information files).
