# Supplementary material for: Drowning in the Eastern Mediterranean region: a systematic literature review of the epidemiology, risk factors and strategies for prevention
Source: BMC Public Health. 2022 Aug 3;22:1477. doi: 10.1186/s12889-022-13778-6 (PMC9351066; doi:10.1186/s12889-022-13778-6)
Supplement: Supplementary file 1 — Additional file 1: Table S1. Search Strategy. Table S2. Description of risk/protective factors, their measure of significance and coding methodology. Table S3. Prevention strategies as described in included studies and coding methodology used, Eastern Mediterranean Region. Table S4. Characteristics of included studies. [file 12889_2022_13778_MOESM1_ESM.pdf]

Supplementary Tables

Table S1: Search Strategy

**Database**	**Search strategy**	**Result**
Embase	1 drown*.mp.	260
	2 submers*.mp.	
	3 immers*.mp.	
	4 2 or 3	
	5 injury.mp or injury/	
	6 4 and 5	
	7 swim*.mp.	
	8 education/ or education.mp.	
	9 7 and 8	
	10 1 o 6 or 9	
	11 Afghan*.mp.	
	12 Bahrain*.mp.	
	13 Djibouti*.mp.	
	14 Egypt*.mp.	
	15 Iran*.mp.	
	16 Iraq*.mp.	
	17 Jordan*.mp.	

18 Kuwait*.mp.

19 Leban*.mp.

20 Libya*.mp.

21 Moroc*.mp.

22 Palestin*.mp.

23 Oman*.mp.

24 Pakistan*.mp.

25 Qatar*.mp.

26 Saudi Arabia.mp. or Saudi Arabia/

27 Somalia*.mp.

28 Sudan*.mp.

29 Syria*.mp.

30 Tunisia*.mp.

31 United Arab Emirates*.mp.

32Yemen*.mp.

33Middle East*.mp.

34 Mediterranean*.mp.

35 11 or 12 or 13 or 14 or 15 or 16 or 17 or 18 or 19 or 20 or 21 or 22 or 23 or 24 or 25 or 26 or 27 or 28 or 29 or 30 or 31 or 32 or 33 or 34

36 10 and 35

PubMed	#1 Search: drown*<br><br>#2 Search: immers* OR submers* AND injury<br><br>#3 Search: swim* AND education<br><br>#4 Search: Afghan* OR Bahrain* OR Djibouti* OR Egypt* OR Iran* OR Iraq* OR Jordan* OR Kuwait* OR Leban* OR Libya* OR Moroc* OR Palestin* OR Oman* OR Pakistan* OR Qatar* OR "Saudi Arabia*" OR Somalia* OR Sudan* OR "Syrian Arab Republic*" OR Tunisia* OR "United Arab Emirates*" OR Yemen* OR Middle East [Mesh]" OR Mediterranean*<br><br>#5 Search: (#1 OR #2 OR #3 AND #4)	416
Scopus	TITLE-ABS-KEY(Drown* OR ((immers* OR submers*) AND injury) AND ("Middle East*" OR Afghan* OR Bahrain* OR Djibouti* OR Egypt* OR Iraq* OR Iran* OR Jordan* OR Kuwait* OR Leban* OR Libya* OR Moroc* OR Palestin* OR Oman* OR Pakistan* OR Qatar* OR "Saudi Arabia*" OR Somalia* OR Sudan* OR Syria* OR Tunisia* OR "United Arab Emirates*" OR Yemen* OR Mediterranean*)) AND (LIMIT-TO (LANGUAGE,"English")) AND (LIMIT-TO (DOCTYPE,"ar") OR LIMIT-TO (DOCTYPE,"re") OR LIMIT-TO (DOCTYPE,"ch"))	536
SportsDiscus	drown* OR ((immers* OR submers*) AND injury) OR (swim* AND education)<br><br>AND<br><br>Afghan* OR Bahrain* OR Djibouti* OR Egypt* OR Iran* OR Iraq* OR Jordan* OR Kuwait* OR Leban* OR Libya* OR Moroc* OR Palestin* OR Oman* OR Pakistan* OR Qatar* OR "Saudi Arabia*" OR Somalia* OR Sudan* OR Syria* OR Tunisia* OR "United Arab Emirates*" OR Yemen* OR "Middle East*" OR Mediterranean*	120
Web of Science	Drown* OR ((immers* OR submers*) AND injury) OR (swim* and education)<br><br>AND<br><br>"Middle East*" OR Mediterranean* OR Afghan* OR Bahrain* OR Djibouti* OR Egypt* OR Iraq* OR Iran* OR Jordan* OR Kuwait* OR Leban* OR Libya* OR Moroc* OR Palestin* OR Oman* OR Pakistan* OR Qatar* OR "Saudi Arabia*" OR Somalia* OR Sudan* OR Syria* OR Tunisia* OR "United Arab Emirates*" OR Yemen*	470
Google Scholar	drown* OR ((immers* OR submers*) AND injury) OR (swim* AND education) AND Afghan* OR Bahrain* OR Djibouti* OR Egypt* OR Iran* OR Iraq* OR Jordan* OR Kuwait* OR Leban* OR Libya* OR Moroc* OR Palestin* OR Oman* OR Pakistan* OR Qatar* OR "Saudi Arabia*" OR Somalia* OR Sudan* OR Syria* OR Tunisia* OR "United Arab Emirates*" OR Yemen* OR "Middle East*" OR Mediterranean*	4 (24 pages of results)

Table S2: Description of risk/protective factors, their measure of significance and coding methodology

Author, year, reference	Country	Study population	Description of risk/protective factor	Measure of significance	Coded risk/protective factor
Al Fifi et al, 2011 (1)	Saudi Arabia	0-13 years	Winter and Autumn	Survival significantly worse (p=0.02)	Seasonality
			Washing containers	Outcome worse than for those who drowned in pools (p=0.04)	Washing containers
			Absence of adult supervision	All fatal drownings were unsupervised by adults (p=0.013)	Adult supervision
			Longer submersion duration	12 minutes median submersion time for fatal vs 3 minutes for non-fatal) (p=0.001)	Submersion time
Alkhalaf et al, 2021 (2)	Saudi Arabia	0-14 years	Delay in initiating resuscitation	A significant association was found between delay in initiating resuscitation and an unfavourable outcome (p<0.01)	Delay in initiating resuscitation
			Submersion time *	A shorter submersion time was significantly associated with a favourable outcome (non-fatal drowning with no neurological damage) (3.5 minutes vs 12.5 minutes) (p<0.01)	Submersion time
			High GCS upon admission *	Complete recovery was associated with high GCS upon admission (p<0.01)	GCS
			Decreased length of stay *	Complete recovery was associated with decreased length of hospital stay (p<0.01)	Length of hospital stay
			High GCS upon end of care *	Complete recovery was associated with high GCS upon the end of care (p<0.01)	GCS

Al-Mofadda et al, 2001 (3)	Saudi Arabia	1-13 years of age	Submersion time	Submersion time >5 minutes predicted fatal outcome (p=0.003)	Submersion time
			Emergency room documentation of absence of vital signs	Emergency room documentation of absence of vital signs significantly associated with brain death or severe neurological disease (p=0.006)	Absence of vital signs
			GCS ≤4	GCS ≤4 associated with brain death or severe neurological disease in 94.1% of cases (p=0.006)	GCS
			Arterial pH of ≤7	Arterial pH of ≤7 associated with brain death or severe neurological disease in 94.1% of cases (p=0.006)	pH level
			Blood sugar of ≥10mmol/L	Blood sugar of ≥10mmol/L associated with brain death or severe neurological disease in 80.0%% of cases (p=0.001)	Blood sugar level
Almuneef et al, 2021 (4)	Saudi Arabia	Children 0-17 years	Age <5 years	Significantly more drowning deaths among children ≤5 years than children >5 years (p<0.01)	Age
			Male gender	Significantly more drowning deaths among children of male gender (p<0.01)	Sex
Al-Thani et al, 2018 (5)	Qatar	Children 1-5 years	Male gender	Males drowned at a rate 2.75 times higher than females (RR=2.75 [95%CI: 1.16-6.50]) (p=0.02)	Sex
Davoudi-Kiakalayeh et al, 2011 (6)	Iran	All age	Male gender	Males were 3.28 times more likely to drown than females	Sex
Davoudi-Kiakalayeh et al, 2013 (7)	Iran	All age	Male gender	Males were 10.9 times more likely to drown than females in the resident population in 2008-09	Sex

Jan et al, 2000 (8)	Saudi Arabia	Parents of infants	Mother with helper or older children leaving infant unattended in bath	Mothers with a helper or older children were more likely to report leaving their infant unattended in the bathtub when compared to those who never left their infant unattended at home (p=0.002)	Mother with helper or older children
Liu et al, 2021 (9)	Bahrain	Children 0-13 years	Age 1-4 years	Drowning cases were significantly more likely to occur among the 1-4 years age group (p<0.001)	Age
Mosayebi et al, 2011 (10)	Iran	Children 0-15 years	Male	Males had a 3.5 times higher odds ratio of fatal drowning than non-fatal drowning when compared to females (OR=3.5 [95%CI:0.38-31.5])	Sex
			Absent vital signs	Significantly associated with mortality (p<0.001)	Absence of vital signs
			GCS<5	Signficantly associated with mortality (p<0.001)	GCS
			Temperature ≥36 degrees *	Positively correlated with recovery (p=0.003)	Body temperature
			pH <7.2	Acidosis associated with unfavourable outcome	pH level
Shahbazi et al, 2020 (11)	Iran	All age	Male sex	Males were more likely to drown than females (p=0.03)	Sex
Vahhab et al, 2019 (12)	Iran	Children and adolescents up to 18 years of age	Age	Children aged 1-4 years more at risk of drowning	Age
			Male sex	Males significantly more at risk than females (p<0.001)	Sex
			Accidental intent	Drowning was more likely to be of accidental intent than due to suicide or homicide (p<0.001)	Accidental intent
			Death at the scene of the incident	Location of death was significantly more likely to be at the scene for drowning, than during transfer to	Death at scene

				hospital, at hospital or after discharge from hospital (p<0.001)	
Zolala et al, 2012 (13)	Iran	All age	Females aged 14 years or less	Females aged 14 years or less were significantly more likely to drown than males of the same age (p=0.03)	Sex<br>Age
			Tourists	Tourists were significantly more likely to drown than local residents (p<0.001)	Resident status
			Local residents	Drowning among local residents was more likely to be fatal than drowning in tourists (p<0.001)	Resident status
			Tourist drowning in the sea	Tourists were significantly more likely than residents to drown in the Caspian Sea (p<0.001)	Resident status
			Non-designated areas (un-lifeguarded)	Drowning was more likely to be fatal in non-designated areas (p<0.001)	Non-lifeguarded areas

* denotes protective factor; GCS-Glasgow Coma Scale

Table S3: Prevention strategies as described in included studies and coding methodology used, Eastern Mediterranean Region

Author, Year, Reference	Study location	Description of prevention strategy	Coded prevention strategy
Abdullah et al, 2013 (14)	Jordan	Establishing a mobile health station on the beach to deal with emergency cases promptly and effectively	CPR and first aid
		Advising people to swim in safe places and only where lifeguards are available (health messages were delivered via signs/signals placed on beach);	Signage
		Assess the safety of beaches and the possibility and efficacy of employing lifeguards	Beach safety assessment
AlAwadhi et al, 2018 (15)	Bahrain	Developing a national water safety strategy is necessary to decrease age-standardized unintentional drowning mortality rate in Bahrain.	Developing a national water safety plan
Al-Fifi et al, 2011 (1)	Saudi Arabia	The media as well as the authority should play a major role in increasing the public awareness to minimize or prevent such a problem.	Public education
		To raise awareness of basic life-support measures (first aid) and the support of emergency medical services.	CPR and first aid
Alkhalaf et al, 2021 (2)	Saudi Arabia	Prevention measures should focus on ensuring that children do not swim unsupervised.	Child supervision
		There is a need to ensure early resuscitation of drowning victims by promoting life support courses and enforcing regulations around water.	CPR and first aid
Davoudi-Kiakalayeh et al, 2011 (6)	Iran	The high-risk population should be trained in basic swimming and rescue skills	Swimming and water safety training

		Many more rescue stations are needed in areas where swimming occurs.	Lifeguard supervision
		Educational campaigns such as integrating public health messages into local television can be employed	Public education
Davoudi-Kiakalayeh et al, 2013 (7)	Iran	Modification of environmental change through, for example, the elimination of certain water reservoirs	Restrict access to water
		Raising community awareness via information programmes for health care workers who employed to educate clients	Public education
		Training high risk populations in basic resuscitation techniques	CPR and first aid
		Integration of public health messages into local television and radio news during the summer season	Public education
		Extension of lifeguard services throughout the beach regions of the Caspian Sea	Lifeguard supervision
		Expansion of the number and scale of rescue service stations in beach areas	Lifeguard supervision
Davoudi-Kiakalayeh et al, 2014 (16)	Iran	Basic swimming skills should be trained to children	Swimming and water safety training
		Additional rescue stations and lifeguarding are needed along the Caspian Sea Coastline (one lifeguard for every 500 meters)	Lifeguard supervision
		Integration of public health messages into local television such as the Darya program is necessary	Public education
		Lake and water canal fencing may help to prevent drowning events in rural settings. The majority of the participants expected that some measures, especially the fencing of rivers and canal water way should be done by governmental organizations	Restrict access to water

Habeeb et al, 2020 (17)	Saudi Arabia	First Aid – describes parental knowledge of first aid for a non-responsive but breathing victim – 64.3% knew to lay the victim in a left lateral position. In a nonresponsive not breathing drowning victim, 90.5% knew to start chest compressions and mouth to mouth ventilation. 65.6% knew not to slap a victim on the back	CPR and first aid
Hammett et al, 2007 (18)	Afghanistan and Iraq	Modifications to vehicles including restraint systems, battle lock modification to vehicle for passengers and rescuers,	Vehicle modification
		Changes to operating procedures when on road near water	Vehicle operation procedures
		Water egress procedure training	Vehicle-related water safety training
Kiakalayeh et al, 2008 (19)	Iran	Should be trained in basic swimming and rescue skills	Swimming and water safety training
		Many more rescue stations are needed in areas where swimming occurs.	Lifeguard supervision
Liu et al, 2021 (9)	Bahrain	Constant adult supervision	Child supervision
		Personal flotation devices	Personal flotation devices
		Swimming education for children over the age of 4	Swimming and water safety training
		Pool isolation fencing	Restrict access to water
Mal et al, 2020 (20)	Pakistan	Strict surveillance and employment of lifeguards at Karachi beaches during Summer	Lifeguard supervision
Mavridou et al, 2014 (21)	Egypt, Lebanon, Palestine and Tunisia	Spa regulations	Spa regulations
		Multi-lingual lifeguards	Lifeguard supervision (multi-lingual lifeguards)
Mlayeh et al, 2021 (22)	Tunisia	Provision of supervision while walking to school for school aged children	Child supervision

Razzak et al, 2013 (23)	Pakistan	Reduce access to water storage systems	Restrict access to water
Ruehsen et al, 1989 (24)	Bahrain	Resuscitation and first aid training should be compulsory for licensed boat owners	CPR and first aid
		All hotels and public swimming areas which are presently unattended should be staffed with a lifeguard	Lifeguard supervision
Shaheen and Edwards, 2008 (25)	Palestine	Health education campaign that targeted parents, using media to highlight problem	Public education
Shaikh, 2014 (26)	Pakistan	Female lifeguards to rescue drowning women	Lifeguard supervision (female lifeguards)
		Police presence at beaches	Lifeguard supervision (police presence at beaches)
Shaikh, 2016 (27)	Pakistan	Need to targeted health education campaigns, especially during summer months	Public education

CPR-Cardio Pulmonary Resuscitation

Table S4: Characteristics of included studies

Author(s), Year, Reference	Country	Year(s) of study	Location	Urban/ Rural/ Both	Study population	Drowning Outcome			Intent			Study Design	NHMRC Level of Evidence
						F	N-F	Un	U	I	Unk		
Abdullah et al, 2013 (14)	Jordan	2010	S	Urban	No age restrictions	✓			✓			Retrospective cross-sectional study	IV
Aghajani et al, 2019 (28)	Iran	2009-2014	N	Both	No age restrictions	✓			✓	✓		Retrospective cross-sectional study	IV
Aghamohamadi et al, 2018 (29)	Iran	2006-2035	N	Both	No age restrictions	✓			✓			Retrospective cross-sectional study	IV
AlAwadhi et al, 2018 (15)	Bahrain	2003-2015	N	Both	No age restrictions	✓			✓			Retrospective descriptive study	IV
Al-Fifi et al, 2011 (1)	Saudi Arabia	1999-2009	S	Urban	Pediatric (0-13 years)	✓	✓				✓	Retrospective cohort study	III-3
Alghnam et al, 2014 (30)	Saudi Arabia	2001-2010	S	Urban	No age restrictions	✓	✓				✓	Retrospective cross-sectional study	IV
Alghnam et al, 2019 (31)	Saudi Arabia	2001-2018	S	Rural	Pediatric (0-18 years)		✓				✓	Retrospective cross-sectional study	IV
Alkhalaf et al, 2021 (2)	Saudi Arabia	2015-2020	N	Both	Child (0-14 years)	✓	✓		✓	✓		Retrospective chart review	IV
Al-Mannai et al, 2014 (32)	Qatar	2010	S	Rural	Child (1-4 years)		✓		✓			Cross-sectional study	IV

Al-Mofadda et al, 2001 (3)	Saudi Arabia	1988-1998	S	Rural	Pediatric (1-13 years)		✓		✓	Retrospective cross-sectional study	IV
Almuneef et al, 2021 (4)	Saudi Arabia	2010-2016	S	Rural	Pediatric (0-18 years)	✓			✓	Retrospective chart review	IV
Al-Qurashi et al, 2019 (33)	Saudi Arabia	2005-2015	S	Rural	Pediatric (0-14 years)		✓		✓	Retrospective cross-sectional study	IV
Alsaif et al, 2018 (34)	Saudi Arabia	1999-2015	S	Rural	Child and adolescent (0-18 years)	✓			✓	Retrospective cross-sectional study	IV
Al-Sammak, 2020 (35)	Iraq	2008-2009 and 2018-2019	S	Rural	No age restrictions	✓		✓		Retrospective cross-sectional study	IV
Al-Talafieh, 1999 (36)	Jordan	1996-1998	S	Both	No age restrictions	✓	✓		V	Retrospective cross-sectional study	IV
Al-Thani et al, 2018 (5)	Qatar	2004-2016	N	Both	Child (1-4 years)	✓		✓		Retrospective cross-sectional study	IV
Anary et al, 2010 (37)	Iran	2002-2006	S	Rural	No age restrictions	✓			✓	Retrospective cross-sectional study	IV
Astaraki et al, 2016 (38)	Iran	2007-2014	S	Rural	Child and adolescent (<18 years)	✓			✓	A descriptive cross-sectional study	IV
Atallah et al, 2008 (39)	Jordan	2014	S	Rural	No age restrictions	✓	✓		✓	Retrospective cross-sectional study	IV

Barss et al, 2009 (40)	UAE	1998-2002	N	Both	No age restrictions	✓					✓	Retrospective cross-sectional study	IV
Barzegar et al, 2019 (41)	Iran	2011-2015	N	Both	No age restrictions	✓				✓		Retrospective cross-sectional study	IV
Bazeli et al, 2017 (42)	Iran	2014-2015	N	Both	No age restrictions	✓	✓		✓			Retrospective cross-sectional study	IV
Bener et al, 1998 (43)	UAE	1995	S	Rural	Child (0-14 years)	✓					✓	Retrospective cross-sectional study	IV
Bener et al, 2011 (44)	Qatar	1993-2007	N	Both	Child (0-18 years)	✓			✓			Retrospective cross-sectional study	IV
Bilukha et al, 2016 (45)	Iraq	2010-2013	N	Both	No age restrictions	✓			✓			Retrospective cross-sectional study	IV
Bozorgi et al, 2016 (46)	Iran	2008-2013	S	Rural	No age restrictions	✓			✓			Retrospective cross-sectional study	IV
Davoudi-Kiakalayeh et al, 2011 (6)	Iran	2007-2008	S	Urban	No age restrictions	✓	✓		✓			Retrospective cross-sectional study	IV
Davoudi-Kiakalayeh et al, 2012 (47)	Iran	2005-2009	S	Urban	No age restrictions	✓			✓			Interventional design (pre- and post-intervention observations)	III-2
Davoudi-Kiakalayeh et al, 2013 (7)	Iran	2005-2008	S	Urban	No age restrictions	✓			✓			A quasi-experimental design	III-2

Davoudi-Kiakalayeh et al, 2014 (16)	Iran	2005-2009	S	Urban	No age restrictions	✓		✓			Register-based cohort study and household survey	III-2
Derakhshan et al, 2020 (48)	Iran	1990-2015	N	Both	No age restrictions	✓				✓	Retrospective cross-sectional study	IV
Elhak et al, 2009 (49)	Egypt	1998-2004	S	Rural	No age restrictions	✓			✓		Retrospective cross-sectional study	IV
Franklin et al, 2020 (50)	EMR	1990-2017	M	Both	No age restrictions	✓		✓			Retrospective cross-sectional study	IV
Ghodsi et al, 2020 (51)	Iran	2015-2016	N	Both	No age restrictions	✓				✓	Retrospective cross-sectional study	IV
Habeeb et al, 2020 (17)	Saudi Arabia	2015	S	Urban	Parents (no age specified <30, 30-40, >40 years)	✓	✓	✓			Cross-sectional survey analysis	IV
Hajebi et al, 2016 (52)	Iran	2009-2012	N	Both	No age restrictions	✓	✓		✓		Retrospective descriptive study	IV
Halawa et al, 2015 (53)	Egypt	2011	N	Both	Child (0-18 years)		✓			✓	Community-based, cross-sectional survey	IV
Hammett et al, 2007 (18)	Afghanistan and Iraq	2003-2005	N	Both	Adult (active duty service members) ages 19-40)	✓		✓			Retrospective descriptive epidemiological study	IV
He et al, 2015 (54)	Pakistan	2010-2011	N	Urban	No age restriction		✓	✓	✓		Retrospective cross-sectional study	IV

Hijazi et al, 2007 (55)	Saudia Arabia	1999-2004	S	Urban	Child (<12 years)		✓				✓	Prospective observational study	III-3
Hooshangi et al, 2020 (56)	Iran	2010-2015	S	Urban	Child (< 5 years)	✓					✓	Cross-sectional secondary alysis of data	IV
Jan et al, 2013 (8)	Saudi Arabia	1999-2000	S	Urban	Parents of infants (infant mean age 4.7 months)	✓	✓		✓			Interviews using a semi-structured questionnaire	IV
Khalil et al, 2013 (57)	Pakistan	2009-2012	S	Urban	No age restrictions	✓					✓	Retrospective cross-sectional study	IV
Khazaei et al, 2020 (58)	Iran	2009-2015	S	Urban	Child (1-5 years)			✓			✓	Retrospective cross-sectional study	IV
Kiakalayeh et al, 2008 (19)	Iran	2005-2006	S	Both	No age restrictions	✓			✓			Retrospective, population-based study	III-3
Kiakalayeh et al, 2011 (59)	Iran	2005-2006	S	Both	No age restrictions	✓			✓	✓		Retrospective cross-sectional study	IV
Lin et al, 2019 (60)	Egypt and Morocco	2004-2005 & 2014-2015	M	Both	No age restrictions	✓			✓			Retrospective, total population study	IV
Liu et al, 2021 (9)	Bahrain	2018 (Sept-Dec)	N	Both	Child (<14 years)	✓	✓				✓	Multicentre observational cross-sectional study	IV
Malik, 1975 (61)	Sudan	1970	N	Both	No age restrictions	✓			✓	✓		Retrospective cross-sectional study	IV

Mal et al, 2020 (20)	Pakistan	2013-2018	S	Both	No age restrictions	✓					✓	Descriptive cross-sectional study	IV
Mavridou et al, 2014 (21)	Lebanon, Palestine, Egypt, Tunisia	Not specified	M	Both	No age restrictions			✓			✓	Cross-sectional survey	IV
Mlayeh et al, 2021 (22)	Tunisia	2008-2015	S	Rural	No age restrictions	✓			✓	✓		Retrospective cross-sectional study	IV
Mokdad et al, 2018 (62)	EMR	1990-2015	M	Both	Adolescents (10-24 years)	✓	✓		✓			Retrospective, total population study	IV
Mosayebi et al, 2011 (10)	Iran	1995-2005	S	Both	Child (0-17 years)	✓	✓				✓	Retrospective cross-sectional study	IV
Naghavi et al, 2010 (63)	Iran	2005	N	Both	Child (0-14 years)	✓	✓		✓			Retrospective cross-sectional study	IV
Pal et al, 2018 (64)	Pakistan	2007-2016	S	Urban	No age restrictions	✓					✓	Retrospective cross-sectional study	IV
Razzak et al, 2013 (23)	Pakistan	2006-2007	N	Both	Child (<5 years)	✓					✓	Retrospective cross-sectional study	IV
Razzak et al, 2004 (65)	Pakistan	1993-1996	S	Rural	Child (≤15 years)	✓	✓				✓	Retrospective, descriptive study	IV
Roudsari et al, 2006 (66)	Iran	1999-2000	S	Both	Child (0-15 years)	✓			✓			Retrospective cross-sectional study	IV
Ruehsen et al, 1989 (24)	Bahrain	1984-1985	S	Both	No age restrictions		✓				✓	Retrospective cross-sectional study	IV

Samaneh et al, 2012 (67)	Iran	2008-2009	S	Urban	No age restrictions	✓			✓	✓		Retrospective cross-sectional study	IV
Shahbazi et al, 2020 (11)	Iran	2013-2018	N	Both	No age restrictions	✓					✓	Descriptive, cross-sectional study	IV
Shaheen and Edwards, 2008 (25)	Palestinian Territory	2001-2003	N	Both	Child (0-19 years)	✓			✓	✓		Retrospective cross-sectional study	IV
Shaikh, 2014 (26)	Pakistan	Not specified	S	Urban	No age restrictions	✓	✓				✓	Cross-sectional survey	IV
Shaikh, 2016 (27)	Pakistan	2012-2014	S	Rural	No age restrictions	✓	✓				✓	Retrospective cross-sectional study	IV
Sheikhazadi et al, 2009 (68)	Iran	2002-2006	S	Rural	No age restrictions	✓			✓	✓		Retrospective cross-sectional study	IV
Siddiqui et al, 2012 (69)	Pakistan	2005-2007	S	Semi-rural	Child (0-14 years)		✓		✓			Retrospective descriptive study	IV
Soori et al, 1999 (70)	Iran	1993-1994	N	Rural	No age restrictions	✓			✓			Retrospective cross-sectional study	IV
Soori et al, 2016 (71)	EMR	2004	N	Both	Child (< 20 years)	✓			✓			Secondary analysis of data	IV
Vahhab et al, 2019 (12)	Iran	2011-2016	S	Urban	Child and adolescent (0-18 years)	✓			✓	✓		Retrospective cross-sectional study	IV
Waseem et al, 2011 (72)	Pakistan	6 months (not specified)	S	Both	No age restrictions			✓			✓	Retrospective case review	IV

Zolala et al, 2012 (13)	Iran	2007-2011	S	Both	No age restrictions	✓	✓		✓			Retrospective cross-sectional study	IV

NHMRC – National Health and Medical Research Council; UAE – United Arab Emirates; EMR-Eastern Mediterranean Region; M-Multi-national, N-National, S-Subnational, F-Fatal, N-F- Non-Fatal, U-Unspecified, Un-Unintentional, I-Intentional, U-Unspecified

References

1. Al-Fifi S, Shabana M, Zayed M, Al-Binali A, Al-Shehri M. Drowning in children: Aseer Central Hospital experience, Southwestern Saudi Arabia. Journal of Family and Community Medicine 2011;18(1):13-6.
2. Alkhalaf H, Zuraie M, Alqahtani Ryan N, Alghamdi M, Afif Abdulrahman B, Alqahtani Faisal J, et al. The epidemiology of drowning among Saudi children: results from a large trauma center. Ann Saudi Med. 2021;41(3):157-64.
3. Al-Mofadda SM, Nassar A, Al-Turki A, Al-Sallounm AA. Pediatric Near Drowning: The Experience of King Khalid University Hospital. 2001;21(5-6):300-3.
4. Almuneef M, Saleheen H, AlBuhairan F, Al-Eissa M, Al Muntaser M, Al Alem H, et al. Child mortality in Saudi Arabia: Time for action at all levels. Int J Pediatr Adolesc Med. 2021;8(3):165-71.
5. Al-Thani M, Al-Thani A-A, Toumi A, Khalifa S, Ijaz MA, Akram H. Trends in the leading causes of childhood mortality from 2004 to 2016 in Qatar. Central Asian journal of global health. 2018;7(1):334-.
6. Davoudi-Kiakalayeh A, Dalal K, Yousefzade-Chabok S, Jansson B, Mohammadi R. Costs related to drowning and near drowning in northern Iran (Guilan province). Ocean and Coastal Management. 2011;54(3):250-5.
7. Davoudi-kiakalayeh A, Mohammadi R, Yousefzade-Chabok S, Jansson B. Evaluation of a community-based drowning prevention programme in northern Islamic Republic of Iran. East Mediterr Health J. 2013;19(7):629-37.
8. Jan MM. Pediatric near-drowning and drowning. Saudi Med J. 2013;34(2):119-22.
9. Liu JC, Ismael AA, Zaidi A, Sha'ban BW, Almutawa SE, Chatha AA, et al. Epidemiology of pediatric trauma in the Kingdom of Bahrain: a national pediatric trauma registry pilot study. Injury Epidemiology. 2021;8(1):42.
10. Mosayebi Z, Movahedian A, Mousavi G. Drowning in children in Iran: outcomes and prognostic factors. Med J Malaysia. 2011;66(3):187-90.
11. Shahbazi F, Mirtorabi SD, Mahdavi SAH, Nazari SSH. Trend of mortality rate due to drowning in Iran (2013-2018). ARCHIVES OF TRAUMA RESEARCH. 2020;9(3):111-5.
12. Vahhab E, Gholamzadeh S, Zarenezhad M. Injury-related pediatric mortality in Shiraz, Iran: A community-based survey. Shiraz E Med J. 2019;20(2).
13. Zolala F, Nasehi MM, Moosazadeh M, Azadeh H, Mirzajani M. Epidemiology of drowning in Mazandaran, Iran: a population based study. Middle-East J Sci Res. 2012;11.
14. Abdullah S, Sharkas G, Sabri N, Iblan I, Abdallat M, Jriesat S, et al. Mass gathering in Aqaba, Jordan, during Eid Al Adha, 2010. East Mediterr Health J. 2013;19.
15. Al Awadhi MA, Abulfateh NM, Board A. Drowning Epidemiology in Bahrain (2003-2015). Bahrain Med Bull. 2018;40(1).
16. Davoudi-Kiakalayeh A, Mohammadi R, Yousefzade-Chabok S. Maternal Beliefs and Socioeconomic Correlated Factors on Child Mortality from Drowning in Caspian Sea Coastline. Bull Emerg Trauma. 2014;2(2):86-91.
17. Habeeb KA, Alarfaj G. Saudi parents awareness regarding burn, choking, and drowning first aid in children. Journal of Family Medicine and Primary Care. 2020;9(3).
18. Hammett M, Pearse L, Naito N, Watts D, Hooper T. Drowning Deaths of U.S. Service Personnel Associated with Motor Vehicle Accidents Occurring in Operation Iraqi Freedom and Operation Enduring Freedom, 2003–2005. Military Medicine. 2007;172(8):875-8.
19. Kiakalayeh AD, Mohammadi R, Ekman DS, Chabok SY, Janson B. Unintentional drowning in northern Iran: A population-based study. Accident Analysis and Prevention. 2008;40(6):1977-81.
20. MAL S, TIRMIZI SZA, WASEEM F, MAKHDOOM PA, IMRAN S, MIRZA FH, et al. Medicolegal Analysis of Fatal Drowning in Karachi: An Autopsy Based Study. PAKISTAN JOURNAL OF MEDICAL & HEALTH SCIENCES. 2020;14(4):1212-5.

21. Mavridou A, Pappa O, Papatzitze O, Blougoura A, Drossos P. An overview of pool and spa regulations in Mediterranean countries with a focus on the tourist industry. Journal of Water and Health. 2014;12(3):359-71.
22. Mlayeh S, Ben Abderrahim S, Haggui F, Ghzel R, Jedidi M. Deadly falls into wells: A retrospective study of 72 autopsy cases from Kairouan, Tunisia. Journal of Forensic Sciences. 2021;66(3):934-9.
23. Razzak JA, Khan UR, Zia N, Azam I. A child an hour: burden of injury deaths among children under 5 in Pakistan. Archives of Disease in Childhood. 2013;98(11):867.
24. Ruehsen MM, Abdul-Wahab A-WM. The Epidemiology of Trauma in an Intensive Care Unit in Bahrain. Journal of Trauma and Acute Care Surgery. 1989;29(1).
25. Shaheen A, Edwards P. Flying bullets and speeding cars: analysis of child injury deaths in the Palestinian Territory. East Mediterr Health J. 2008;14(2):406-14.
26. Shaikh MA. Risks of drowning and safety concerns at the beaches of Karachi--perspective from lifeguards. The Journal of the Pakistan Medical Association. 2014;64(5):576-8.
27. Shaikh MA. Epidemiology of drowning and near drowning at Karachi beaches from 2012 to 2014. The Journal of the Pakistan Medical Association. 2016;66(5):602-5.
28. Aghajani MH, Haddadi M, Saadat S. Epidemiological pattern of injuries in iran; A nationwide review of seven million emergency department admissions. Arc Acad Emer Med. 2019;7(1).
29. Aghamohamadi S, Jahangiri K, Kavousi A, Sayah Mofazali A. Analysis and Forecasting the Accident Mortality Trends in the Islamic Republic of Iran Applying Lee-Carter Model During the Years 2006 to 2035. Bulletin of emergency and trauma. 2018;6(4):341-8.
30. Alghnam S, Alkelya M, Al-Bedah K, Al-Enazi S. Burden of traumatic injuries in Saudi Arabia: lessons from a major trauma registry in Riyadh, Saudi Arabia. Ann Saudi Med. 2014;34(4):291-6.
31. Alghnam S, Towhari JA, Al Babtain I, Al Nahdi M, Aldebasi MH, Alyami M, et al. The associations between injury mechanism and extended hospital stay among pediatric patients: findings from a trauma Center in Saudi Arabia. BMC Pediatrics. 2019;19(1):177.
32. Al-Mannai L, Selim NAA, Al-kuwari MG, Ismail MFS. Unintentional injuries among preschool children in Qatar: Cross sectional study. World Family Medicine Journal. 2014;12(1):36-44.
33. Al-Qurashi FO, Yousef AA, Aljoudi A, Alzahrani SM, Al-Jawder NY, Al-Ahmar AK, et al. A Review of Nonfatal Drowning in the Pediatric-Age Group: A 10-Year Experience at a University Hospital in Saudi Arabia. Pediatric Emergency Care. 2019;35(11).
34. Alsaif DM, Almadani OM, Ali Almoghannam S, Hamdi D, Al-Farayedhi MA, Kharosha MA. Child Fatalities in Dammam: A Call for Child Fatality Reviews in Arab Countries. Journal of Forensic Sciences. 2018;63(4):1171-5.
35. Al-Sammak NI. Types of Accidents in Nineveh Governorate/Iraq. International Journal of Psychosocial Rehabilitation. 2020;24(05).
36. Al Talafieh A, Al Majali R, Al Dehayat G. Clinical, laboratory and X-ray findings of drowning and near-drowning in the Gulf of Aqaba. East Mediterr Health J. 1999;5(4):706-9.
37. Anary SHS, Sheikhazadi A, Ghadyani MH. Epidemiology of Drowning in Mazandaran Province, North of Iran. AMERICAN JOURNAL OF FORENSIC MEDICINE AND PATHOLOGY. 2010;31(3):236-42.
38. Astaraki P, Mahmoudi G, Ahadi M, Almasi V. The reasons of death among children and adolescents in Lorestan Province, west of Iran, from 2007 to 2014. International Journal of Medical Toxicology and Forensic Medicine. 2016;6(2):65-70.
39. Atallah AI. Epidemiology of trauma injuries in the districts of Amman, Jordan: problems and suggestions. JEMTAC - Journal of Emergency Medicine, Trauma and Acute Care 2008;8(1):35-7.
40. Barss P, Subait OM, Ali MHA, Grivna M. Drowning in a high-income developing country in the Middle East: Newspapers as an essential resource for injury surveillance. Journal of Science and Medicine in Sport. 2009;12(1):164-70.

41. Barzegar A, Ghadipasha M, Aram S, Khademi A. Suicides, homicides, and fatal occupational accidents in Iran: A cross-sectional, retrospective study based on autopsy reports; need to develop preventive strategies. Iranian Red Crescent Medical Journal. 2019;21(4):e65672.
42. Bazeli J, Aryankhesal A, Khorasani-Zavareh D. Epidemiology of special incidents: Results from national mortality and morbidity registry and the associated factors in Iran in 2014. Electronic physician. 2017;9(8):5113-21.
43. Bener A, Al-Salman KM, Pugh RNH. Injury mortality and morbidity among children in the United Arab Emirates. Eur J Epidemiol. 1998;14(2):175-8.
44. Bener A, Hussain SJ, Ghaffar A, Abou-Taleb H, El-Sayed HF. Trends in childhood trauma mortality in the fast economically developing State of Qatar. World J Pediatrics. 2011;7(1):41-4.
45. Bilukha OO, Sultan ASS, Hassan A, Hussain SJ, Leidman E. Injury-related fatalities in selected governorates of Iraq from 2010 to 2013: Prospective surveillance. Am J Disaster Med. 2016;11(1):49-58.
46. Bozorgi F, Derakhshanfar H, Hoseininejad SM, Abbasi A, AbolghasemLali MT. Cogitation on Routine Drowned Cases in Mazandaran Province: a CaseStudyinbetweentheYear2008to2013. Ambient Science. 2016;3(2):61-3.
47. Davoudi-Kiakalayeh A, Mohammadi R, Yousefzadeh-Chabok S. Prevention of Drowning by Community-Based Intervention: Implications for Low- and Middle-Income Countries. ARCHIVES OF TRAUMA RESEARCH. 2012;1(3):112-7.
48. Derakhshan P, Saeedi Moghaddam S, Saadat S, Ghanbari A, Rezaei N, Khosravi S, et al. Trends in the drowning mortality rate in Iran. Injury Prevention. 2020;26(4):351-9.
49. Gad ElHak SA, El-Ghazali AM, Salama MM, Aboelyazeed AY. Fatal suicide cases in Port Said city, Egypt. Journal of Forensic and Legal Medicine. 2009;16(5):266-8.
50. Franklin RC, Peden AE, Hamilton EB, Bisignano C, Castle CD, Dingels ZV, et al. The burden of unintentional drowning: global, regional and national estimates of mortality from the Global Burden of Disease 2017 Study. Injury Prevention. 2020;26(Supp 1):i83-i95.
51. Ghodsi Z, Saadat S, Barzegar A, Alaeddini F, Rahimi-Movaghar V, Zafarghandi M, et al. The completeness of the registration system and the economic burden of fatal injuries in Iran. Turkish Journal of Trauma and Emergency Surgery. 2020;26(5):671-7.
52. Hajebi A, Ahmadzad-Asl M, Davoudi F, Ghayyomi R. Trend of suicide in Iran during 2009 to 2012: Epidemiological evidences from national suicide registration. Iran J Psychiatr Behav Sci. 2016;10(4).
53. Halawa EF, Barakat A, Rizk HII, Moawad EMI. Epidemiology of non-fatal injuries among Egyptian children: a community-based cross-sectional survey. BMC Public Health. 2015;15(1):1248.
54. He S, Lunnen JC, Zia N, Khan UR, Shamim K, Hyder AA. Pattern of presenting complaints recorded as near-drowning events in emergency departments: a national surveillance study from Pakistan. BMC Emergency Medicine. 2015;15(2):S4.
55. Hijazi OM, Shahin AA, Haidar NA, Sarwi MF, Musawa ES. Effect of submersion injury on water safety practice after the event in children, Saudi Arabia. Saudi Med J. 2007;28(1):100.
56. Hooshangi Z, Salehi S, Amini-Rarani M. Explanatory social variables of under-5 mortality caused by injury in Isfahan Province, Islamic Republic of Iran. East Mediterr Health J. 2020;26(6):660-7.
57. Khalil ZH, Naeem M, Adil M, Khan MZ-u-I, Abbas SH. Analysis of autopsy record of unnatural deaths in Peshawar district. J Postgrad Med Inst. 2013;27(4).
58. Khazaei S, Ayubi E, Sanaei Z, Jenabi E, Khazaei L, Amiri J. Epidemiologic characteristics of injuries among 1-5 year-old children in Hamadan Province: Analysis of 4523 hospitalized children over a 6-year period. Archives of Trauma Research. 2020;9(1):3-7.
59. Kiakalayeh AD, Mohammadi R, Ekman DS, Yousefzade-Chabok S, Behboudi F, Jansson B. Estimating drowning deaths in Northern Iran using capture-recapture method. Health Policy. 2011;100(2-3):290-6.

60. Lin C-Y, Wang L-Y, Lu T-H. Changes in drowning mortality rates and quality of reporting from 2004–2005 to 2014–2015: a comparative study of 61 countries. BMC Public Health. 2019;19(1):1391.
61. Malik MOA. Some Aspects of the Mortality Pattern in the Sudan. Med Sci Law. 1975;15(4):246-60.
62. Azzopardi P, Cini K, Kennedy E, Sawyer S, El Bcheraoui C, Charara R, et al. Adolescent health in the Eastern Mediterranean Region: findings from the global burden of disease 2015 study. International Journal of Public Health. 2018;63(1):79-96.
63. Naghavi M, Pourmalek F, Shahraz S, Jafari N, Delavar B, Motlagh ME. The burden of injuries in Iranian children in 2005. Popul Health Metr. 2010;8(1):5.
64. Pal MI, Qasim AP, Naeem M, Arshad H, Saeed A. Profile of Asphyxial Deaths in Faisalabad: A 10-Year Study. J Coll Phys Surg Pak. 2018;28:266+.
65. Razzak JA, Luby SP, Laflamme L, Chotani H. Injuries among children in Karachi, Pakistan—what, where and how. Public Health. 2004;118(2):114-20.
66. Roudsari BS, Shadman M, Ghodsi M. Childhood trauma fatality and resource allocation in injury control programs in a developing country. BMC Public Health. 2006;6(1):117.
67. Samaneh A, Samaneh A, Hamid S, Ardeshir K, Khosro G. Years of potential life lost due to unintentional drowning mortality in Mazandaran province, Iran. World Appl Sci J. 2012;20(10):1433-8.
68. Sheikhazadi A, Ghadyani MH. Epidemiology of drowning in Isfahan province, center of Iran. J Res Med Sci. 2009;14(2):79-87.
69. Siddiqui E, Ejaz K, Siddiqui U. Unintentional, paediatric domestic injury in a semi rural area of Karachi. Journal of Pakistan medical association. 2012;62(7):638.
70. Soori H, Naghavi M. Deaths from unintentional injuries in rural areas of the Islamic Republic of Iran. East Mediterr Health J. 1999;5(1):55-60.
71. Soori H, Khodakarim S. Child unintentional injury prevention in Eastern Mediterranean Region. International Journal of Critical Illness and Injury Science. 2016;6(1):33-9.
72. Waseem H, Carenzo L, Razzak J, Naseer R. Epidemiology of major incidents: an EMS study from Pakistan. Int J Emer Med. 2011;4(1):48.
